# Mechanisms of Antidiabetic Activity of Methanolic Extract of *Punica granatum* Leaves in Nicotinamide/Streptozotocin-Induced Type 2 Diabetes in Rats

**DOI:** 10.3390/plants9111609

**Published:** 2020-11-19

**Authors:** Shinu Pottathil, Parminder Nain, Mohamed A. Morsy, Jaspreet Kaur, Bandar E. Al-Dhubiab, Sandhya Jaiswal, Anroop B. Nair

**Affiliations:** 1 Department of Biomedical Sciences, College of Clinical Pharmacy, King Faisal University, Al-Ahsa 31982, Saudi Arabia; 2 Department of Pharmacology, M.M. College of Pharmacy, Maharishi Markandeshwar (Deemed to be University), Mullana-Ambala 133207, India; parminder.nain26@gmail.com (P.N.); preetisidana@gmail.com (J.K.); 3 Department of Pharmaceutical Sciences, College of Clinical Pharmacy, King Faisal University, Al-Ahsa 31982, Saudi Arabia; momorsy@kfu.edu.sa (M.A.M.); baldhubiab@kfu.edu.sa (B.E.A.-D.); anair@kfu.edu.sa (A.B.N.); 4 Department of Pharmacology, Faculty of Medicine, Minia University, El-Minia 61511, Egypt; 5 Department of Pharmaceutics, Punjab Technical University, Jalandhar 144603, India; sandhya_jais@rediffmail.com

**Keywords:** *Punica granatum*, antidiabetic, antioxidant, insulin

## Abstract

The current study aimed to establish the mechanisms of antidiabetic activity of methanolic extract of *Punica granatum* leaves (MEPGL) in nicotinamide/streptozotocin-induced type 2 diabetes in rats. Phytochemical screening, HPLC analysis, and acute toxicity study of MEPGL were carried out. Various concentrations of MEPGL (100, 200, 400, and 600 mg/kg) were administered orally to diabetic rats for 45 days on a daily basis. The antidiabetic effect of MEPGL was examined by measuring blood glucose, plasma insulin, and glycated hemoglobin (HbA1c) levels, as well as with an oral glucose tolerance test. The antioxidant effect of MEPGL was determined by analyzing hepatic and renal antioxidant markers, namely superoxide dismutase (SOD), catalase (CAT), glutathione peroxidase (GPx), reduced glutathione (GSH), and lipid peroxidation. The other biochemical markers alanine transaminase (ALT), aspartate transaminase (AST), alkaline phosphatase (ALP), urea, and creatinine, as well as total cholesterol, triglycerides, and high-density lipoprotein (HDL) were also studied. Type 2 diabetes significantly altered these parameters, while oral administration of the MEPGL significantly ameliorated them. Moreover, the pancreatic histopathological changes were attenuated with MEPGL treatment. In a nutshell, oral MEPGL administration in diabetic rats showed antidiabetic activity due to its antioxidant activity, most probably due to the gallic acid, ellagic acid, and apigenin found in MEPGL.

## 1. Introduction

Diabetes mellitus (DM) is a group of metabolic alterations that not only affect carbohydrate metabolism but protein and fat metabolisms as well. A recent report about the prevalence of DM across the globe estimates that ~463 million people are currently affected by DM. This report also proposes the figure may rise up to 10.2% (578 million by 2030) and 10.9% (700 million by 2045), respectively [1]. DM usually develops due to deficiency or lack of insulin secretion or may be also due to the diminished ability of the cells to utilize the insulin [2]. DM is considered to be the most important predisposing factor for the development of various clinical conditions such as ischemic heart diseases, peripheral neuropathies, ulcerations, and delayed wound healings, consequently affecting the life expectancy of the patients [3]. Various experimental and clinical studies suggest the involvement of free radicals in the progression of DM and its complications [4,5,6]. The damage of β-cells of the pancreas may be caused due to the formation of free radicals and/or alternation of their endogenous scavengers [7].

Effective treatment of DM is considered to be an important task for the medical community because most of the currently available drugs have various adverse effects [8]. Various studies have analyzed the alternatives for the treatment of DM that have similar therapeutic efficacy to that of conventional therapeutic agents but without causing significant adverse effects [8,9,10]. However, no alternative treatment options have been recommended to date for treating DM. Ethno-botanical knowledge includes ~1200 species of medicinal plants that have the potential to treat DM and its complications [9]. Several plant extract-based medicines have been practiced for the management of different diseases in Asian countries as well as in different parts of the world. However, the exact mechanisms of the mode of action of these extracts are not well studied. Currently, scientists are evaluating the use of different herbal extracts for treating various diseases including DM [10,11].

*Punica granatum* L. (pomegranate) is a fruiting plant that grows in the subtropics and warm temperate areas. The medicinal use of pomegranate fruit is mentioned in ancient Egyptian literature and traditional Ayurvedic medicine [12]. Various parts of the pomegranate plant (such as pomegranate juice, peel or rind, flower, leaves, and bark) possess marked antioxidant, anti-inflammatory, and immunomodulatory properties, and hence they have antidepressant, antiulcer, anthelminthic, antibacterial, antifungal, antiviral, anticancer, and antidiabetic activities [12,13,14,15]. Various parts of the pomegranate plant contain different types of phytochemicals that possess various pharmacological activities. The leaves of pomegranate are abundant with tannins (punicalin and punicafolin), flavones (luteolin and apigenin), and glycosides [16]. The extracts obtained from different parts of the *Punica granatum* have been studied in the treatment of various ailments. An earlier study has suggested that the methanolic extract of *Punica granatum* leaves (MEPGL) might have an antidiabetic effect [17]. Nevertheless, the mechanisms by which MEPGL exerted this effect were not well-recognized. Different from the earlier study, we have carried out a comprehensive investigation on the possible mechanisms of the antidiabetic activity of MEPGL in the nicotinamide/streptozotocin-induced type 2 diabetes model. Additionally, in the current study, various biochemical and antioxidant (in liver and kidneys) parameters, as well as histopathological changes in the pancreas were evaluated. The literature suggests that the treatment of rats with streptozotocin alone damages pancreatic β-cells resulting in inadequate production of insulin and thereby these animals manifest with type 1 DM [18]. However, nicotinamide partly protects β-cells of the pancreas via nitric oxide-mediated mechanisms and thereby partly preserves the pancreatic β-cells. Therefore, rats that are treated with nicotinamide/streptozotocin produce type 2 DM [19]. Consequently, the current study aimed to explore the potential mechanisms by which MEPGL protects against nicotinamide/streptozotocin-induced type 2 DM in rats.

## 2. Results

The yield of the methanol extract was 7.19%. The dried extract was suspended in 1% aqueous carboxymethyl cellulose to make different concentrations of methanolic extracts before administration into test rats.

### 2.1. Phytochemical Screening

The phytochemical screening detected the presence of tannins, glycosides, carbohydrates, flavonoids, and phytosterols in the MEPGL (Table 1).

### 2.2. High Performance Liquid Chromatography (HPLC)

**Figure 1:**
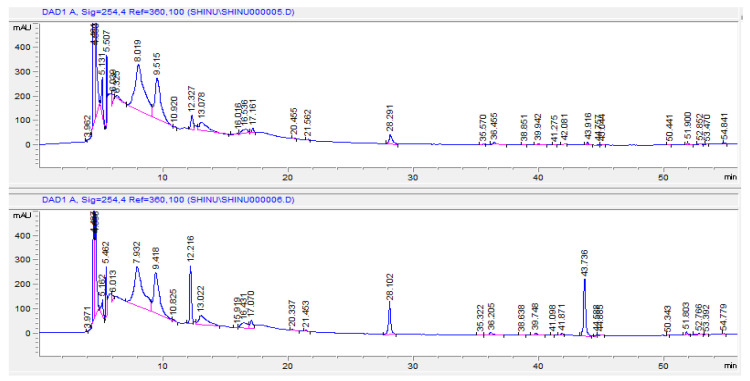
A comparison of the sample chromatogram of methanolic extract of Punica granatum leaves (upper panel) with the chromatogram of sample spiked with standard solutions of gallic acid, ellagic acid, and apigenin (lower panel).

Two phenolic acids (gallic acid and ellagic acid) and one flavonoid (apigenin) from the standard solutions were eluted at 12.2, 28.1, and 43.7 min, respectively. The comparison of the sample chromatogram with the chromatogram of sample spiked with standard solutions of gallic acid, ellagic acid, and apigenin confirms the presence of these compounds in the MEPGL (Figure 1; upper and lower panels). The chromatogram showed many other peaks indicating the presence of various other constituents.

### 2.3. Acute Toxicity Study of MEPGL

The acute oral toxicity of MEPGL showed no toxic signs even after 24 h of extract administration. Further, no oral toxicity or mortality was detected even after oral administration of higher doses (up to 2000 mg/kg) of MEPGL for 21 days (one dose per day). This indicates the safety of the extract for prolonged use.

### 2.4. Effect of MEPGL on Body Weight

The rats treated with MEPGL (Groups 4–7) showed significant increase (*p* < 0.05) in their body weight in a similar manner with that of normal control rats (Table 2).

### 2.5. Effect of MEPGL on Blood Glucose and Plasma Insulin Levels

In normal control rats, no changes in the levels of fasting blood sugar were noted. On the contrary, in diabetic rats, the blood glucose levels increased significantly while plasma insulin levels significantly decreased suggesting an inverse relationship between blood glucose and plasma insulin levels during the course of the experiment. In contrast, a significant (*p* < 0.05) reduction in blood glucose levels was observed when diabetic rats were treated with glibenclamide or MEPGL (except Group 4 on the 22nd day). On the other hand, a considerable (*p* < 0.05) increase in plasma insulin levels was noted in diabetic rats when treated with glibenclamide or MEPGL (except Group 4 on the 22nd day) (Table 3).

### 2.6. Effect of MEPGL on Oral Glucose Tolerance Test (OGTT)

**Figure 2:**
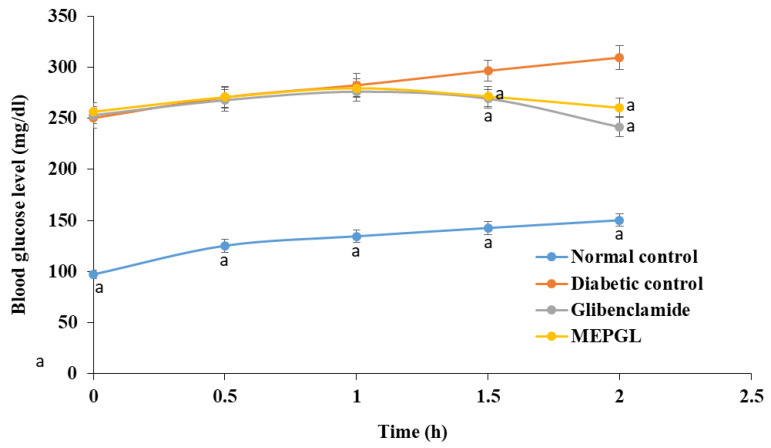
Effect of methanolic extract of Punica granatum leaves (600 mg/kg) on oral glucose tolerance test in nicotinamide/streptozotocin-induced type 2 diabetes in rats. Data are given as mean ± SEM (*n* = 6). ^a^ Significant (*p* < 0.05) difference compared to the diabetic group.

After 120 min of oral intake of MEPGL (maximum dose of 600 mg/kg) and glibenclamide (dose of 1 mg/kg), blood glucose levels were considerably (*p* < 0.05) decreased in comparison with diabetic rats (Figure 2).

### 2.7. Effect of MEPGL on Different Biochemical Parameters

The hepatic function of diabetic rats was studied in terms of alanine transaminase (ALT), aspartate transaminase (AST), and alkaline phosphatase (ALP). Similarly, renal function was studied by estimating urea and creatinine levels. On the other hand, lipid profile was determined in terms of total cholesterol, triglycerides, and high-density lipoprotein (HDL). Consecutive oral intake of MEPGL for 45 days resulted in a significant (*p* < 0.05) reduction in ALT (Groups 6–7), creatinine (Group 7), and triglycerides (Groups 4–7), as well as a significant increase in HDL (Group 7) compared to the diabetic group (Table 4).

### 2.8. Effect of MEPGL on Oxidant–Antioxidant Status in Liver and Kidney

Enzymatic (superoxide dismutase (SOD), catalase (CAT), glutathione peroxidase (GPx)) and non-enzymatic (reduced glutathione (GSH)) antioxidants, as well as lipid peroxidation (expressed as malondialdehyde (MDA)) analyses were used for estimating the oxidative stress that was induced in the diabetic rats. A significant (*p* < 0.05) rise in MDA and a significant fall in the measured antioxidants were noted in parenchymal cells of both liver and kidney. In contrast, oral administration of MEPGL considerably ameliorated these changes in a dose-dependent manner and even restored the normal values at the highest used concentration (600 mg/kg) (Table 5 and Table 6).

### 2.9. Effect of MEPGL on Pancreatic Histopathology

**Figure 3:**
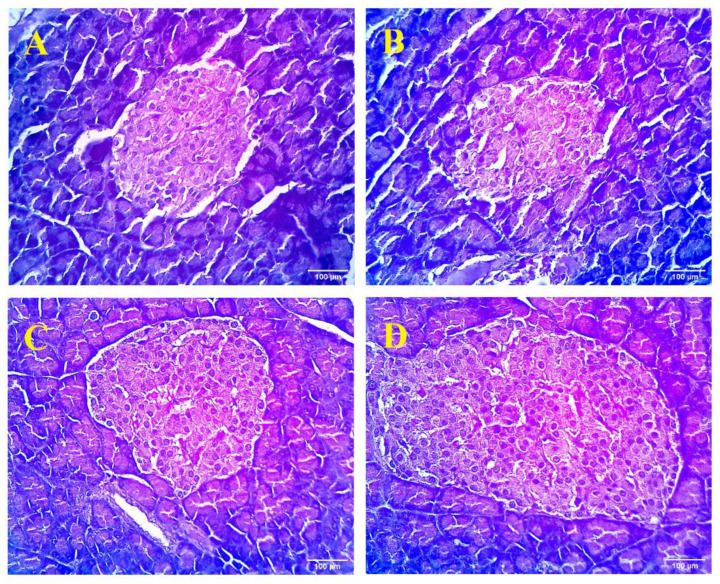
Light microscopic examination of pancreatic tissue (stained by hematoxylin and eosin stain) obtained from various experimental groups. (**A**): Normal group showing normal appearance of the pancreas; (**B**): Diabetic group revealed pathological changes in parenchymal cells of pancreatic tissue; (**C**): Diabetic rats treated with glibenclamide displaying restoration of the general architecture; and (**D**): Diabetic rats treated with Punica granatum (600 mg/kg) showing nearly normal architecture of pancreas.

The histological examination of the pancreas of the normal rats illustrated the normal architecture of islets of Langerhans, which were evenly distributed in the pancreatic tissue with different sizes in the same lobule of the pancreas (Figure 3). Each islet was arranged in anastomosing cellular plates and a reticular membrane was separated from each acinus. Alternatively, the pancreas of the diabetic control group revealed the presence of peripheral widening between the islets of Langerhans and pancreatic acini. In the glibenclamide-treated group, Langerhans cells were densely arranged, with insignificant space between adjacent cells and the absence of inflammatory cells. Architectural disarray was noted in the pancreas to some extent when compared to diabetic control animals. However, diabetic rats that underwent treatment with MEPGL (dose of 600 mg/kg) showed relatively less pronounced architectural changes and low peripheral broadening between acinar and Langerhans cells as compared with diabetic rats. Overall, MEPGL (600 mg/kg) showed maximum recovery from all histopathological changes with a partial proliferation of β-cells.

## 3. Discussion

Humans have been using herbal medicines for thousands of years. Ayurveda and Unani, the two indigenous systems of alternative medicines, document many crude drug preparations for the management of various diseases. The formulations of these medicines contain a rich legacy of herbal extracts [20]. Various plant species have been part of folk medicine in different cultures and are used as a treatment option against DM across the world [21]. Although different oral and systemic antidiabetic agents are available in the market, the need for natural antidiabetic products is increasing as a complementary remedy [10,22].

Literature indicates that the rats that are treated with nicotinamide/streptozotocin produced type 2 DM, but rats treated with streptozotocin alone induced type 1 DM [18,19,23,24]. Streptozotocin selectively damages the insulin-secreting β-cells of the pancreas and thereby produces a diabetic condition. The insufficient level of insulin further results in the disability of cells to use glucose and subsequently results in the production of reactive oxygen species (ROS) [25]. However, nicotinamide partially protects pancreatic β-cells against streptozotocin by inhibition of poly (adenosine diphosphate ribose-ribose) polymerase-1 activity and serves as a precursor of nicotinamide adenine dinucleotide [18]. Furthermore, these experimental rats demonstrate various diabetic complications, such as cardiomyopathy, retinopathy, nephropathy, and neuropathy, which particularly develop through oxidative stress-induced mechanisms [26]. The results from the current investigation show a substantial reduction in fasting plasma insulin levels of diabetic rats. These results are similar to the characteristic findings of nicotinamide/streptozotocin-induced type 2 DM in rats [18]. The findings of the current study demonstrate a considerable enhancement of insulin concentration with a significant decline in fasting glucose levels in MEPGL-treated diabetic rats. The hypoglycemic effect of MEPGL may be attributed to its role in restoring the physical state of the cellular plasma membrane and its related functions such as glucose transport, which is essentially under insulin control [27]. Moreover, the antioxidant property of MEPGL might have a role in increasing insulin levels by protecting the β-cells of the pancreas against oxidative stress-induced cellular injury [28].

Body weight reduction due to the imbalance of metabolic pathways is commonly associated with DM [29]. In the current study, diabetic rats treated with MEPGL significantly gained weight, most likely due to reversing the glycogenolysis and gluconeogenesis and thereby helping the restoration of normal metabolic pathways [30]. In diabetic rats, a low total hemoglobin level was noted, mainly due to the excessive production of glycated hemoglobin (HbA1c). The excess blood glucose combines with the globin part of the hemoglobin to produce elevated levels of HbA1c; therefore, decreasing hemoglobin levels in the blood. This indicates a direct relationship between the HbA1c levels and the blood glucose level [31,32]. In diabetic rats, the oral administration of MEPGL significantly increased the hemoglobin level in addition to a significant reduction in the HbA1c level, indicating the potential of MEPGL to establish normal glycemic activity. On the other hand, an abnormal lipid profile was observed in the diabetic rats when compared to the normal group. This may be because of the imbalance in the various metabolic and regulatory pathways developed that are mainly due to the deficiency of insulin [33].

The elevation in liver enzymes is characteristically associated with the glycemic status in type 2 diabetic patients [34]. In this study, various liver enzymes such as ALT, AST, and ALP were elevated in diabetic rats. The elevated transaminases might contribute to the evolution of diabetic ketogenesis and gluconeogenesis [35]. Alternatively, in diabetic rats, MEPGL treatment significantly reduced liver transaminases activity. This indicates that MEPGL may act as a hepatoprotective agent in diabetes. In renal dysfunction that is induced by diabetic hyperglycemia, the serum urea and creatinine levels are markedly elevated [36]. In this study, the diabetic rats showed elevated serum creatinine and urea levels suggesting the impairment of the kidney in filtering the toxic or waste products out of the body. Furthermore, the serum creatinine and urea levels were considerably diminished in MEPGL-treated diabetic rats, suggesting a renoprotective effect on diabetic rats.

In DM, autoxidation of glucose results in the production of ROS that further enhance lipid peroxidation and produce lipoxidation end products and more free radicals [28]. Lipid peroxidation is accountable for protein aggregation, which causes liver damage and vascular complications of DM [37]. Hyperglycemia is coupled with a rise in plasma MDA, a lipid peroxidation product and a marker of free radical production. In experimental models of DM, high levels of lipid peroxidation in both hepatic and renal parenchymal cells were noticed [38], which are in line with the findings of the present study. The findings of the current study illustrate that treatment with MEPGL considerably lowers the level of lipid peroxidation and minimizes the likelihood of tissue injury.

In type 2 DM, the raised levels of ROS might result in a hypercoagulable state and the evidence indicates that the oxidation products accumulate earlier than the development of DM [39]. Hyperglycemia diminishes the antioxidant defenses. For example, in harmony with the present study, streptozotocin-treated rats showed a low concentration of hepatic and renal SOD, CAT, and GPx [40]. SOD, CAT, and GPx play an imperative function in the elimination of free radicals from the tissues. In the current study, the diabetic rats treated with MEPGL demonstrated a substantial rise in hepatic and renal SOD, CAT, and GPx activities. This finding clearly shows the role of MEPGL, particularly its flavonoid and tannin contents, in improving the antioxidant levels and thereby suppressing the action of free radicals. In the current study, the GSH levels in diabetic rats were found to be significantly lower when compared to normal rats. This can be ascribed to the increased level of the free radical generation that converts more GSH to its oxidized form [41]. In the current study, diabetic rats treated with MEPGL showed an increase in hepatic and renal GSH as compared to control rats. This suggests that the MEPGL may either enhance the production of GSH and/or may decrease the oxidative stress resulting in a reduction in GSH levels. The protection of GSH is by scavenging the free radicals, acting as a cofactor for antioxidant enzymes, and accelerating the xenobiotic detoxification [42]. GPx reacts with various lipid hydroperoxides that are generated during lipid peroxidation and it enhances the reduction of hydrogen peroxide in the presence of GSH to water. SOD and CAT also participate in the metabolism of hydrogen peroxide [43]. However, hepatic and renal GPx activity were significantly enhanced by the administration of MEPGL.

The photomicrograph of the diabetic pancreatic tissue clearly shows the streptozotocin-induced damage in both exocrine and endocrine components of pancreatic tissue. Glibenclamide stimulates the pancreatic islets regeneration and is responsible for the increase in the plasma insulin as observed during biochemical evaluations and the histological photomicrograph [44]. It was observed that the MEPGL showed protective activity against ROS-mediated damage, which occurs in the islets of Langerhans cells of the pancreas.

The phytochemical analysis of MEPGL illustrates the existence of polyphenols (for instance tannins, glycosides, and flavonoids), phytosterols, and carbohydrates. Literature indicates that medicinal herbs that possess both antidiabetic and antioxidant activity mostly contain high concentrations of polyphenols such as tannins and flavonoids, steroid glycosides, and terpenoids [45,46]. The rind flower and seeds of *Punica granatum* demonstrate antioxidant and antidiabetic activity particularly attributed to polyphenols such as gallic acid, ellagic acid, and ascorbic acid [12]. In the current study, consistent with the earlier findings [47,48], HPLC analysis of MEPGL revealed the existence of gallic acid, ellagic acid, and apigenin. Gallic acid has an antidiabetic effect by inhibiting the functioning of α-amylase and α-glucosidase, important enzymes linked to type 2 DM [49]. Alternatively, it is known that ellagic acid has its antidiabetic effect partly by enhancing the glucose utilization of the peripheral and adipose tissues [50], as well as by stimulating β-cells of the pancreas to secrete insulin [51]. Moreover, apigenin showed an antidiabetic effect by enhancing glucose metabolism via decreasing oxidative stress [52]. The antidiabetic and antioxidant properties of MEPGL may be attributed to either single or synergistic action of the above phytoconstituents.

## 4. Materials and Methods

### 4.1. Chemicals

Streptozotocin was procured from Sigma-Aldrich (St. Louis, MO, USA). Glibenclamide was a gift from Wockhardt (Mumbai, Maharashtra, India). All other used reagents were of analytical grade.

### 4.2. Animals

Male Wistar rats aged 7–8 weeks (200 ± 10 g) were obtained from the animal house at MM College of Pharmacy, Maharishi Markandeshwar (deemed to be a university), Ambala, India. The animals were accommodated in groups of six rats each and were kept at 25 ± 1 °C temperature and 45–55% relative humidity, with 12:12 h light/dark cycle. Animals were freely allowed to move for food and water. The acclimatization of animals with laboratory environments was done prior to the beginning of the experiment (at least one week). The experimental protocol was approved (protocol no 585/05/A/CPCSEA) by the institutional ethics committee and the study was performed as per the Indian National Science Academy Guidelines for the use and care of experimental animals.

### 4.3. Preparation of the MEPGL

Leaves of Punica granatum were obtained from the National Park (Yamunanagar, Haryana, India) in the month of June. Identification and confirmation of the plant material were performed (ref no NISCAIR/RHMD/CONSULT/-2010/1336/138) at National Institute of Science Communication and Information Resources (New Delhi, India). The leaves were shade dried for 20 days and then crushed in a grinder to make a coarse powder. The extract was obtained by mixing coarse powder leaves (250 g) with methanol (500 mL) in a Soxhlet apparatus at a temperature of 65 °C and the process was continued until the siphoning solution became colorless. The filtration and concentration of the extract were performed using a rotary evaporator. The dried extract is dark green in color and it was stored in an airtight container at −80 °C for preliminary phytochemical screening and biochemical analysis.

### 4.4. HPLC Analysis

#### 4.4.1. Preparation of Standards and Samples for HPLC

Standard solutions of gallic acid, ellagic acid, and apigenin were prepared by dissolving 10 mg of each in 10 mL of methanol. Sample solutions were prepared by dissolving MEPGL in 50% methanol and 0.1% trifluoroacetic acid and spiked with standard solutions. Ten microliters of samples were injected into the HPLC system for the analysis.

#### 4.4.2. Chromatographic Conditions

The HPLC system (Agilent Technologies, 1200 series; Tokyo, Japan) comprised of a quaternary gradient pump, an auto-sampler to inject samples, a built-in degasser, and a diode array detector was used for the identification of analytes in the extract. The separation of analytes and other extract constituents was achieved with a Zorbax TMS (250 mm × 4.6 mm, 5 µm, i.d.) column by gradient elution at 254 nm. The mobile phase consists of a mixture of 0.1% trifluoroacetic acid (solvent A) and acetonitrile (solvent B), which was allowed to flow at a rate of 0.7 mL/min at the ambient temperature. The gradient elution program started with solvent A and solvent B in a ratio of 90:10 followed by 60:40 till 28 min, 40:60 till 39 min, and 10:90 till 60 min.

### 4.5. Phytochemical Screening of MEPGL

The MEPGL was subjected to qualitative analysis of various phytoconstituents including alkaloids, tannins, glycosides, carbohydrates, reducing sugars, saponins, steroids, flavonoids, and phytosterols as described in the literature [53].

### 4.6. Acute Toxicity Study

Acute oral toxicity of Punica granatum leaves was conducted in experimental animals as per Organisation for Economic Co-operation and Development-423 (acute toxic class method) guidelines. Healthy female Wistar rats (nulliparous and non-pregnant) were randomly distributed into groups with five rats in each group. The overnight fasting of animals (8–10 h) was achieved by providing them with only water. After that, MEPGL was administered via oral route; a single dose of different concentrations (5, 50, 300, and 2000 mg/kg p.o.) to determine the safe doses based on a stepwise procedure [54]. These rats were continually monitored for 1 h, repeatedly for 4 h, and at 24 h for common signs of toxicity. Within 24 h, the mortality rate was recorded for each group and the surviving rats were observed on daily basis for a further 14 days for signs of delayed toxicity and lethality. The log-probit analysis was used to estimate the lethal dose 50% lethal dose 50% (LD_50_) [55]. No post-mortem studies were carried out during the toxicity test.

### 4.7. Induction of Type 2 DM

Type 2 DM was produced in fasted rats as described previously [56]. A single intraperitoneal (i.p.) administration of streptozotocin (60 mg/kg of body weight was dissolved in 0.1 mol/L cold citrate buffer), 15 min after i.p. administration of nicotinamide (120 mg/kg), was given to the rats. The desirable hyperglycemia (fasting blood glucose level > 200 mg/dL) was checked using a hem glucometer (Abbott, Chicago, IL, USA) after 72 h of nicotinamide/streptozotocin injection.

### 4.8. Experimental Design and Sample Collection

For the current study, 42 rats were separated into seven different groups (*n* = 6). These include the normal control group (Group 1); diabetic control group (Group 2); diabetic rats treated with glibenclamide (1 mg/kg) (Group 3) [57]; diabetic rats given MEPGL (100, 200, 400, and 600 mg/kg, respectively) (Groups 4–7). Rats not treated with streptozotocin/nicotinamide or MEPGL received their vehicles in the same volume and route of administration.

After 45 days of diabetes induction, overnight-fasted rats were euthanized, and blood was removed to obtain plasma and serum. In addition, the liver and kidney were rapidly removed, cleaned (using ice-cold saline), homogenized (in 0.25 M sucrose and 0.1 M Tris-HCl buffer solution, pH 7.4), centrifuged (at 3000 rpm for 10 min), and the supernatant was used for detection of various oxidative stress biomarkers. At the same time, pancreatic tissues were carefully excised for histopathological examination.

### 4.9. Biochemical Analysis

Blood glucose and plasma insulin levels were quantified after collecting blood from the eye orbital sinus of overnight-fasted rats on the first day, 22nd day, and 45th day after elicitation of diabetes. For the OGTT, oral administration of aqueous glucose solution (2 g/kg body weight) was administered 30 min after glibenclamide and MEPGL treatments. Blood samples were obtained at 0, 30, 60, 90, and 120 min for blood glucose level estimation. The biochemical parameters, namely hemoglobin, glycated hemoglobin (HbA1c), ALT, AST, ALP, urea, creatinine, total protein, total cholesterol, triglycerides, HDL, and oxidative stress parameters (SOD, CAT, GPx, GSH, and lipid peroxidation) were also determined. The radioimmunoassay method was used for the assay of plasma insulin levels [58]. Standard methods were used to estimate the level of SOD [59], CAT [60], GPx [61], GSH [62], and lipid peroxidation [63] in the liver and kidney. The other biochemical tests were performed using kits from Erba Diagnostics (Miami, FL, USA).

### 4.10. Histopathological Examination

The pancreas was removed, and the tissues were washed in ice-cold normal saline immediately after sacrificing the animal. The tissues were fixed in 10% formal saline for 24 h to avoid decomposition. Afterward, the tissues were cleaned and embedded in paraffin wax (melting point 58–60 °C). Sectioning of the paraffin-embedded pancreas (at 7 µm) was performed using a semi-automated microtome. Then, a hot plate was used to mount the tissue sections on a glass slide. The tissue sections were subjected to deparaffinization and dehydration using xylene and alcohol, respectively. Finally, the tissue sections were stained using hematoxylin and eosin stain. The slides were microscopically examined (under 400× magnification) by a pathologist using a light microscope (BX43, Olympus, Japan) equipped with a digital camera.

### 4.11. Statistical Analysis

The results are analyzed in terms of mean ± SEM. All data were further analyzed using one-way analysis of variance (ANOVA) followed by the least significant difference test. *p* values < 0.05 were considered as statistically significant.

## 5. Conclusions

The current study establishes the hypoglycemic and antioxidant properties of MEPGL and, therefore, offers credence to its folkloric use in the treatment and/or control of type 2 DM. The leaves of the plant contain several chemical substances that are capable of producing different types of pharmacological activities using various mechanisms. Therefore, it is challenging to depict a rational conclusion on the mode of action. However, evidence from the present study suggests that the MEPGL via its antioxidant effect protects tissues from damage induced by oxidative stress. The antidiabetic effect of MEPGL, possibly due to gallic acid, ellagic acid, and apigenin, is also most probably partly due to its antioxidant activity. However, the hypoglycemic effect due to either rise in insulin production by stimulating the pancreatic β-cells or by enhancing the peripheral cellular glucose uptake cannot be ruled out.

## Figures and Tables

**Table 1:** Phytochemical screening of the methanolic extract of Punica granatum leaves.

Chemical Test	Observation	Inference
**Alkaloids**		
(i) Dragendorff test	Reddish precipitate were not observed	Alkaloids absent
(ii) Hager test	Yellow precipitate were not observed	Alkaloids absent
(iii) Mayer test	A milky coloration was not observed	Alkaloids absent
**Tannins**		
(i) Bromine water test	The bromine water was decolorized	Tannins present
(ii) Ferric chloride test	A blue black precipitate was observed	Tannins present
**Glycosides**		
(i) General test for glucoside	Formation of yellow color	Glycosides present
**Carbohydrates**		
(i) Molisch test	Formation of red color at the interphase of two layers	Carbohydrates present
(ii) Fehling test	Not Formation of a red precipitate	Reducing sugar present
**Saponins**		
(i) Froth test	No significant frothing was obtained	Saponins absent
**Steroids**		
(i) Acetic anhydride test	A bluish green interphase was not obtained	Steroids absent
(ii) Liebermann-Burchard test	Pink to red color not formed	Steroids absent
**Flavonoids**		
(i) Magnesium ribbon test	Effervescence was occurred	Flavonoids present
(ii) Shinoda test	Magenta color was appear	Flavonoids present
**Phytosterols**		
(i) Salkowski test	Black coloration was observed	Phytosterols present

**Table 2:** Effect of the methanolic extract of Punica granatum leaves (100, 200, 400, and 600 mg/kg; Groups 4–7) on body weight in nicotinamide/streptozotocin-induced type 2 diabetes in rats.

Day	Body Weight (g)						
	Group 1	Group 2	Group 3	Group 4	Group 5	Group 6	Group 7
1st day	206 ± 5.5	198 ± 3.0	210 ± 4.5	212 ± 5.5	207 ± 6.0	205 ± 5.0	200 ± 5.5
22nd day	245 ± 4.5 ^a^	180 ± 5.0	240 ± 5.0 ^a^	205 ± 6.5	219 ± 5.5 ^a^	227 ± 6.5 ^a^	239 ± 6.0 ^a^
45th day	290 ± 6.0 ^a^	155 ± 5.5	284 ± 7.0 ^a^	209 ± 6.0 ^a^	233 ± 5.0 ^a^	251 ± 6.5 ^a^	268 ± 5.0 ^a^

Data are denoted as mean ± SEM (*n* = 6). ^a^ Significant (*p* < 0.05) difference compared to the diabetic group (Group 2).

**Table 3:** Effect of methanolic extract of Punica granatum leaves (100, 200, 400, and 600 mg/kg; Groups 4–7) on blood glucose and plasma insulin levels in nicotinamide/streptozotocin-induced type 2 diabetes in rats.

Group	Blood Glucose (mg/dL)			Plasma Insulin (μU/mL)		
	1st Day	22nd Day	45th Day	1st Day	22nd Day	45th Day
Group 1	91.3 ± 3.10	92.0 ± 3.60	92.4 ± 3.90	17.8 ± 0.73	17.1 ± 0.80	18.5 ± 0.61
Group 2	271 ± 3.20 ^a^	310 ± 4.90 ^a^	353 ± 4.10 ^ab^	9.21 ± 0.92 ^a^	6.43 ± 0.81 ^a^	5.31 ± 0.76 ^a^
Group 3	280 ± 3.80 ^a^	190 ± 4.10 ^ab^	98.6 ± 3.80 ^b^	8.90 ± 0.70 ^a^	12.5 ± 0.67 ^ab^	16.6 ± 0.79 ^b^
Group 4	277 ± 3.0 ^a^	247 ± 4.50 ^ac^	222 ± 4.30 ^abc^	9.89 ± 1.03 ^a^	9.32 ± 0.91 ^ac^	9.65 ± 1.03 ^ab^
Group 5	282 ± 3.40 ^a^	233 ± 4.30 ^abc^	190 ± 5.20 ^abc^	8.97 ± 1.07 ^a^	9.79 ± 1.04 ^abc^	10.9 ± 1.13 ^abc^
Group 6	278 ± 3.20 ^a^	212 ± 5.10 ^abc^	142 ± 4.70 ^abc^	9.03 ± 1.11 ^a^	10.4 ± 1.19 ^ab^	12.2 ± 1.08 ^ab^
Group 7	283 ± 3.80 ^a^	188 ± 4.70 ^ab^	107 ± 3.80 ^b^	8.80 ± 1.28 ^a^	12.0 ± 1.14 ^ab^	14.7 ± 1.16 ^ab^

Data are denoted as mean ± SEM (*n* = 6). ^a^ Significant (*p* < 0.05) difference compared to the normal group (Group 1). ^b^ Significant (*p* < 0.05) difference compared to the diabetic group (Group 2). ^c^ Significant (*p* < 0.05) difference compared to the glibenclamide-treated group (Group 3).

**Table 4:** Effect of methanolic extract of Punica granatum leaves (100, 200, 400, and 600 mg/kg; Groups 4–7) on different biochemical parameters in nicotinamide/streptozotocin-induced type 2 diabetes in rats.

Parameters	Group 1	Group 2	Group 3	Group 4	Group 5	Group 6	Group 7
Hemoglobin (mg/dL)	13.2 ± 3.2	8.57 ± 2.60 ^a^	12.1 ± 2.90 ^b^	9.42 ± 1.30 ^a^	10.0 ± 1.50	11.3 ± 1.70	12.1 ± 1.30 ^b^
HbA1c (%)	5.67 ± 0.70	13.1 ± 2.40 ^a^	6.91 ± 1.09 ^b^	13.0 ± 0.97 ^ac^	11.6 ± 0.89	9.30 ± 0.90	7.15 ± 1.10 ^b^
ALT (U/L)	27.2 ± 2.80	54.5 ± 3.80 ^a^	29.7 ± 3.70 ^b^	46.6 ± 3.20 ^ac^	39.8 ± 3.90	32.7 ± 3.70 ^b^	28.3 ± 3.80 ^b^
AST (U/L)	45.3 ± 4.0	87.9 ± 5.70 ^a^	48.2 ± 4.60 ^b^	80.5 ± 5.90 ^ac^	64.7 ± 4.90 ^b^	53.4 ± 5.50 ^b^	49.7 ± 5.10 ^b^
Alkaline phosphatase (U/L)	124 ± 5.10	280 ± 5.50 ^a^	143 ± 7.10 ^b^	260 ± 4.30 ^ac^	202 ± 4.90 ^abc^	161 ± 5.10 ^ab^	138 ± 4.70 ^b^
Urea (mg/dL)	32.0 ± 3.0	78.9 ± 3.20 ^a^	39.6 ± 2.40 ^b^	77.6 ± 2.60 ^ac^	63.4 ± 2.20 ^abc^	51.3 ± 1.70 ^abc^	41.2 ± 2.50 ^b^
Creatinine (mg/dL)	0.98 ± 0.24	2.85 ± 0.21 ^a^	1.03 ± 0.30 ^b^	2.19 ± 0.30	1.83 ± 0.40	1.39 ± 0.50	1.11 ± 0.60 ^b^
Total protein (g/dL)	7.90 ± 0.70	4.80 ± 0.35 ^a^	8.00 ± 0.82 ^b^	5.80 ± 0.46	6.60 ± 0.71	7.50 ± 0.53	8.20 ± 0.88 ^b^
Total cholesterol (mg/dL)	135 ± 6.10	263 ± 6.70 ^a^	180 ± 6.30 ^b^	240 ± 5.0 ^ac^	199 ± 7.10 ^abc^	171 ± 4.20 ^ab^	150 ± 5.30 ^b^
Triglycerides (mg/dL)	78.4 ± 2.70	166 ± 4.10 ^a^	87.6 ± 3.40 ^b^	150 ± 3.60 ^abc^	130 ± 3.40 ^abc^	110 ± 3.10 ^abc^	86.3 ± 3.90 ^b^
HDL (mg/dL)	49.9 ± 3.10	24.8 ± 3.90 ^a^	39.9 ± 3.20 ^b^	21.7 ± 3.30 ^ac^	28.7 ± 2.90 ^a^	36.5 ± 4.20	42.5 ± 4.40 ^b^

Data are denoted as mean ± SEM (*n* = 6). ^a^ Significant (*p* < 0.05) difference compared to the normal group (Group 1). ^b^ Significant (*p* < 0.05) difference compared to the diabetic group (Group 2). ^c^ Significant (*p* < 0.05) difference compared to the glibenclamide-treated group (Group 3). HbA1c: glycated hemoglobin; ALT: alanine transaminase; AST: aspartate transaminase; HDL: high-density lipoprotein.

**Table 5:** Effect of methanolic extract of Punica granatum leaves (100, 200, 400, and 600 mg/kg; Groups 4–7) on hepatic oxidant–antioxidant parameters in nicotinamide/streptozotocin-induced type 2 diabetes in rats.

Group	Liver				
	SOD (unit/mg Protein)	CAT (μmol/min/mg Protein)	GPx (μmol/min/mg Protein)	GSH (mM/100 mg Tissue)	MDA (μmol/100 mg Tissue)
Group 1	9.06 ± 1.12	91.8 ± 3.11	11.3 ± 1.09	59.6 ± 2.25	1.07 ± 0.54
Group 2	4.90 ± 0.86 ^a^	42.4 ± 2.23 ^a^	5.44 ± 0.90 ^a^	25.6 ± 1.88 ^a^	2.12 ± 0.60 ^a^
Group 3	5.89 ± 1.76 ^a^	63.1 ± 4.65 ^ab^	8.24 ± 2.31 ^ab^	44.3 ± 2.50 ^a^	1.97 ± 0.32 ^a^
Group 4	5.24 ± 0.45 ^a^	39.6 ± 2.56 ^a^	6.75 ± 1.34 ^a^	30.6 ± 2.00 ^a^	1.91 ± 0.68 ^a^
Group 5	6.50 ± 0.32 ^ab^	50.4 ± 3.67 ^a^	7.87 ± 1.58 ^a^	36.5 ± 2.56 ^a^	1.65 ± 0.51 ^a^
Group 6	7.27 ± 0.40 ^ab^	67.7 ± 3.93 ^ab^	8.70 ± 1.55 ^ab^	41.3 ± 2.78 ^ab^	1.37 ± 0.76 ^ab^
Group 7	8.86 ± 0.37 ^b^	83.2 ± 3.56 ^b^	10.7 ± 1.93 ^b^	50.4 ± 2.52 ^b^	1.14 ± 0.45 ^b^

Data are given as mean ± SEM (*n* = 6). ^a^ Significant (*p* < 0.05) difference compared with the normal group (Group 1). ^b^ Significant (*p* < 0.05) difference compared to the diabetic group (Group 2). SOD: superoxide dismutase; CAT: catalase; GPx: glutathione peroxidase; GSH: reduced glutathione; MDA: malondialdehyde.

**Table 6:** Effect of methanolic extract of Punica granatum leaves (100, 200, 400, and 600 mg/kg; Groups 4–7) on renal oxidant–antioxidant parameters in nicotinamide/streptozotocin-induced type 2 diabetes in rats.

Group	Kidney				
	SOD (unit/mg Protein)	CAT (μmol/min/mg Protein)	GPx (μmol/min/mg Protein)	GSH (mM/100 mg Tissue)	MDA (μmol/100 mg Tissue)
Group 1	15.2 ± 0.72	70.6 ± 3.61	12.4 ± 1.98	35.3 ± 1.95	1.90 ± 0.56
Group 2	9.11 ± 0.59 ^a^	45.4 ± 3.95 ^a^	6.7 ± 1.08 ^a^	21.4 ± 1.19 ^a^	3.03 ± 0.93 ^a^
Group 3	12.8 ± 0.87 ^b^	60.5 ± 4.11 ^b^	10.5 ± 0.92 ^b^	32.2 ± 1.78 ^b^	2.80 ± 0.31 ^a^
Group 4	10.8 ± 0.79 ^a^	46.5 ± 3.43 ^a^	8.00 ± 1.09 ^a^	23.9 ± 1.28 ^a^	2.94 ± 0.76 ^a^
Group 5	11.2 ± 0.81 ^ab^	61.8 ± 3.18 ^a^	8.05 ± 1.10 ^a^	27.9 ± 1.56 ^ab^	2.80 ± 0.83 ^a^
Group 6	12.8 ± 0.87 ^b^	66.6 ± 3.72 ^b^	10.1 ± 1.89 ^b^	30.9 ± 1.96 ^b^	2.76 ± 0.48 ^ab^
Group 7	14.6 ± 0.62 ^b^	70.5 ± 4.17 ^b^	12.2 ± 1.65 ^b^	33.8 ± 1.87 ^b^	2.01 ± 0.59 ^b^

Data are given as mean ± SEM (*n* = 6). ^a^ Significant (*p* < 0.05) difference compared to normal group (Group 1). ^b^ Significant (*p* < 0.05) difference compared to the diabetic group (Group 2). SOD: superoxide dismutase; CAT: catalase; GPx: glutathione peroxidase; GSH: reduced glutathione; MDA: malondialdehyde.
